# Influence of Different Lactation Stages on Circadian Rhythmicity of Metabolic Biomarkers in Dairy Cows: A Pilot Study

**DOI:** 10.3390/ani11041043

**Published:** 2021-04-07

**Authors:** Anna Mareike Couperus, Fabian Schroeder, Robert Klukas, Johann Huber, Thomas Wittek, Johannes R. Peham

**Affiliations:** 1 Molecular Diagnostics, Center for Health and Bioresources, AIT Austrian Institute of Technology, 1210 Vienna, Austria; johannes.peham@ait.ac.at; 2 Institute of Statistics and Mathematical Methods in Economics, Vienna University of Technology, 1040 Vienna, Austria; Fabian.Schroeder@tuwien.ac.at; 3 University Clinic for Ruminants, University of Veterinary Medicine Vienna, 1210 Vienna, Austria; klukasr@googlemail.com (R.K.); Johann.Huber@vetmeduni.ac.at (J.H.); thomas.wittek@vetmeduni.ac.at (T.W.)

**Keywords:** circadian rhythm, lactation cycle, biomarker monitoring, energy metabolism, metabolic disease, dairy cow, BHB, NEFA

## Abstract

**Simple Summary:**

Circadian rhythms are present in most species and play an important role in their metabolism. Metabolic biomarkers, which are commonly used to assess the health status, are also affected by those rhythms. In this study, we investigate the influence of lactation and time on four metabolic biomarkers in dairy cows. Our findings provide new insights into the physiology of circadian rhythms in dairy cows, which enables novel metabolic monitoring approaches.

**Abstract:**

Currently, subclinical metabolic imbalances at the individual cow and herd level are detected by measuring biomarkers in single blood samples. However, diurnal variations have not been fully described yet but need to be considered when sampling for a robust ad consistent analysis. The study describes the influence of lactation phases on circadian rhythms and diurnal variations for non-esterified fatty acids (NEFA), beta-hydroxybutyrate (BHB), total bilirubin (tBIL) and aspartate aminotransferase (AST) in dairy cows. In an observational pilot study, we used 16 clinically healthy Simmental dairy cows subdivided in four different lactation stages (dry-off, fresh, high and late lactating). Every cow was monitored for 24 h, with blood sampling and assessment of clinical parameters every 2 h. Time and lactation stage influence the concentration of the biomarkers NEFA, BHB and tBIL in serum. Further, circadian rhythmicity was found in high lactating cows for NEFA peaking at 5:39 am and BHB peaking at 4:20 pm. We suggest blood sampling for single-point measurements within three hours after the first feeding until two hours after the last feeding of the day. The results provide a new insight into the physiology of circadian rhythms in dairy cows and enable improved metabolic monitoring.

## 1. Introduction

Dairy cows undergo various metabolic and physiological changes during the course of the different lactation stages. The biological cycles of milk production and reproduction play decisive roles in this [1]. In particular, cows during the transition period from dry-off to early lactation face numerous challenges. Periparturient feed intake depression and endocrine changes associated with parturition and lactogenesis contribute to a state of negative energy balance (NEB) [2,3]. NEB is naturally compensated by mobilization of body fat reserves. However, excessive or imbalanced mobilization has been linked with decreased fertility and several comorbidities, such as ketosis, fat cow syndrome, displaced abomasum, subacute ruminal acidosis and milk fever [4,5,6,7]. Two strategies to test for metabolic diseases are testing herds and monitoring individuals. Both strategies distinguish how the test results are interpreted [8]. Individual results are compared to a normal range [9], whereas the interpretation of herd-based test results is based on the mean of the tested-subgroup or on the proportion of cows above or below a defined cut-off value within the tested subgroup [8,10]. However, both strategies have disadvantages. For individuals only a single timepoint measurement is usually performed, whereas in herd-based management, decisions rely on a relatively small sample set. Especially for threshold diseases such as subclinical ketosis, this is very challenging, since diurnal variations of metabolic biomarkers are often not considered by these approaches. Individual monitoring has become more relevant since cow-side tests for various biomarkers became widely available. Common biomarkers for metabolic monitoring are non-esterified fatty acids (NEFA), beta-hydroxybutyrate (BHB), total bilirubin (tBIL) and aspartate aminotransferase (AST). NEFA are synthetized during lipolysis and levels increase with a NEB. NEFA concentration is an indicator of fat mobilization and NEB [11]. A high NEFA concentration in blood is associated with an increased risk for metabolic diseases [12], while recommended cut-off levels vary with a lactation phase ranging from 0.15 to 0.62 mmol/L [9]. Elevated BHB levels are part of the normal adaptive response after calving. However, excessive concentrations of BHB are indicative of ketosis and considered as a gateway condition for other metabolic diseases [13,14,15]. Cut-off levels of BHB associated with undesirable outcomes have been published for different lactation stages. The recommended BHB level ranges from 0.62 to 1.6 mmol/L for subclinical ketosis [9,16,17,18,19]. tBIL is a product of the hemoglobin catabolism and biliary metabolized. An elevated concentration (>5.3 µmol/L) can be related to an increased NEFA concentration, to a decreased bile flow and to an impaired liver function [9] and thereby be used as a marker for metabolism, hepatocyte dysfunction and damage [20]. AST activity (>80 IU/L) serves as a marker for cell integrity and an increased serum activity is caused by damaged tissue and hepatic lesions [9,21,22]. Diurnal patterns and circadian rhythms in various biomarkers of diverse species have been described in a multitude of studies [23]. Moreover, several zeitgebers which regulate the body’s circadian rhythms have been identified, such as light/dark cycle, activity and feed intake [24]. However, to study circadian rhythmicity in a cohort, the tested individuals must be synchronized in terms of the light/dark cycle, rest/activity schedule and feeding [25]. Postprandial changes and the effect of sampling time on NEFA and BHB were reported in dairy cows by several authors [26,27,28,29]. However, to our knowledge, the influence of different lactation cycles on the variation of biomarkers throughout the day has not been described yet. So far, circadian sampling was done across different physiological stages of production or provided suggestions for all lactation stages based on the results of a few tested stages.

The aim of this study was to identify and compare these lactation specific biomarker time courses and to find an ideal sampling time for herd and individual health monitoring. Our main hypothesis is that the stage of lactation affects diurnal variation of biomarkers and needs to be considered to achieve reliable and meaningful measurement results.

## 2. Materials and Methods

### 2.1. Animals, Study Design, Housing and Diets

This pilot study was performed at the teaching and research dairy farm of the Vetmeduni Vienna (VetFarm Kremesberg, Pottenstein, Austria) from 15 February to 1 March 2016 with a mean monthly temperature of 5.8 °C [30]. A hybrid (linked cross-sectional) chronobiology study design was chosen. A total of 16 clinically healthy, adult female Simmental cows were enrolled in this study. The cows were distributed to four groups (*n* = 4) according to their lactation status: fresh (0–8 weeks post partum (p.p.), mean sampling point 4.5 weeks p.p.), high (8–25 weeks p.p., mean sampling point 14.3 weeks p.p.), late lactating (>25 weeks p.p., mean sampling point: 34.8 weeks p.p.) and dry (<8 weeks ante partum (a.p.), mean sampling point 4.5 weeks a.p). The cows were between their 1st to 9th lactation (mean lactation number 3.13, standard deviation (SD) = 2.60). The average 305 days milk yield of the enrolled cows was 7203 kg (SD = 1808 kg), lying within national average of Simmental dairy herds in Austria (7220 kg in 2015 [31]). A total of 2 cows and 3 cows were successfully bred back in the high lactating group and in the late lactating group, respectively. All cows were kept under identical management and housing conditions and were thereby synchronized in terms of housing, feeding, milking and rest-activity schedule. Lights were out from 12:00 pm to 5:00 am. Neither the feeding nor the milking regime was changed in the course of the study. Lactating cows were fed a total mixed ration consisting of 19.23% hay, 16.67% grass silage, 44.23% corn silage, 11.41% grain mix and 8.46% of a protein supplement (RINDASTAR 39 XP; H. Wilhelm Schaumann GmbH & Co KG, Brunn am Gebirge, Austria). Dry cows were fed with a dry-cow total mixed ration consisting of 24.25% hay, 9.33% grass-silage, 46.08% maize-silage, 12.13% barley-straw and 8.21% of a protein supplement (RINDASTAR 39 XP; H. Wilhelm Schaumann GmbH & Co KG, Brunn am Gebirge, Austria)). Feed was mixed and delivered by an automatic mixing and feeding system (Triomatic T15, Trioliet Feeding Technology, Oldenzaal, The Netherlands). Fresh feed was offered to lactating cows ad libitum 11 times per day (between 4.50 am and 9.30 pm). Dry cows were offered fresh feed ad libitum twice per day (7.15 am and 4 pm), respectively. All cows were housed in a free stall barn with straw bedding and had continuous access to water basins and mineral stones (Raiffeisenverband Salzburg reg.Gen.m.b.H, Salzburg, Austria) throughout the study. Twice a day (6.30 am and 4.30 pm), cows were milked in a 4 × 4 tandem milking parlor (DeLaval GmbH Eugendorf, Austria).

### 2.2. Data Collection

Every cow was monitored for 24 h, with sampling every other hour (*n* = 13). The first and final samples was taken at 8.00 am.

#### 2.2.1. Blood Sampling

The blood was collected from the jugular vein using a permanent intravenous catheter (Intraflow 2 (PTFE) 80 mm × 2.7 mm, Vygon, Ecouen, France) or a vacuum tube system (Vacuette^®^, Greiner Bio-one International, Kremsmünster, Austria) using 10 mL serum vacutainer tubes with coagulant (Vacuette^®^ Z Serum Clot Activator, Greiner Bio-one International, Kremsmünster, Austria). The permanent intravenous catheter was inserted 24 h prior to the first sample withdrawal. When the catheter became non-functional, the vacuum tube system was used (21 out of 208 samples). Here, the cows were restrained using head gates and a chain was used to hold off the jugular vein. All blood samples were kept at room temperature for 2 h to allow clotting. The serum was separated by centrifugation at 3000× *g* for 15 min. Samples were stored at −80 °C until analysis which was performed within a maximum of 8 weeks.

#### 2.2.2. Clinical Parameters

To monitor the clinical health status, indication of stress and to check whether behavioral and metabolic synchronization was achieved, rumen contractions, heart rate and chewing activity were assessed prior to each sampling. Heart rate and rumen contractions were determined by auscultation. Additionally, the rectal temperature was recorded with a digital thermometer (MT1831, Microlife, Widnau, Switzerland) every 6 h. In case of an elevated rectal temperature (>39 °C), the measurement was repeated every other hour as well. The chewing activity was assessed visually. Severe changes in the behavioral patterns and pathological elevated parameters were exclusion criteria for the study.

#### 2.2.3. Milk Data

Milk yields were electronically recorded by the milking parlor (DeLaval GmbH Eugendorf, Austria).

### 2.3. Serum Analysis

NEFA, BHB, tBIL and AST activity were analyzed with an automatic analyzer for clinical chemistry (Cobas 6000/c501; Roche Diagnostics GmbH, Vienna, Austria) using standardized colorimetric enzymatic assays. NEFA was analyzed with the ASC-ACOD method (Wako Chemicals, Richmond, VA, USA, inter-day coefficient of variability (CV) < 0.75%, inter-day CV at 0.55 mmol/L = 0.75%, inter-day CV at 1.08 mmol/L = 4.91%). BHB was determined using the Ranbut method (Randox Laboratories Ltd., London, UK, inter-day CV = 0.57%, intra-day CV = 0.99%). tBIL was measured with the Bilirubin Total DPD Gen.2 Kit (Roche Diagnostics GmbH, Vienna, Austria, inter-day CV = 1.6%, intra-day CV = 2.6%). AST activity was analyzed by kinetic measurement of the enzyme activity with pyridoxal phosphate activation recommended by the International Federation of Clinical Chemistry (Roche Diagnostics GmbH, Vienna, Austria, inter-day CV = 0.6%, intra-day CV = 0.8%). All measurements were performed at the Clinical Pathology Platform of the Vetmeduni Vienna.

### 2.4. Statistical Analysis

All data were analyzed with R (Version 3.0.4). The time series of each parameter was analyzed by visual inspection of a 24 h’ time plot. The effects of lactation stages and time were further assessed by a multilevel linear model based on a repeated measurements design with a three-level hierarchy, in which observations (level 1) were nested within cows (level 2), which were nested within groups corresponding to their lactation stage (level 3). Data obtained from the same cow were considered as repeated measurements. Lactation stage and time were considered as fixed effects. Cow was considered as random effects. The normality of all biomarkers (response variables) was tested by means of Shapiro–Wilk tests. Due to the skewness of data, we applied a Log-transformation prior to all analysis. The baseline model was a linear mixed-effects model fit by maximum likelihood and included no predictors other than the intercept. To see the overall effect of both predictors, each was added successively to the baseline model and evaluated separately. All three models (baseline, group and time) were compared by the means of a one-way ANOVA. To analyze the main effects of the lactation phase, a post-hoc Tukey’s pairwise comparison of the lactation group means was performed. The significance level was set to *p* < 0.05. Box and whisker plots were used to depict the distribution of the four lactation groups. To determine the significancy of the biomarkers’ daily maxima or minima compared to the mean, a one sample *t*-test was performed. Data of biomarkers which showed significant time dependence were further assessed by cosinor based rhythmicity analysis. A cosine curve with a given period was fitted to the data by the least squares method [32]. This method is suitable to analyze short and sparse data series [24,25]. First the cosine curve was fitted for each cow individually and then summarized across all cows within a group [33]. Each of the fitted curves is described by the rhythm-adjusted mean M (Midline Estimating Statistic of Rhythm-MESOR), the amplitude A (half the predictable variation within a cycle), and the acrophase ϕ (time point of predicted maximum of a cycle). The cosine fit was evaluated by an *F*-test. Further, the strength of rhythmicity was calculated. The rhythmicity (R^2^) is defined as the percentage of viability in the data that is accounted by the fit (percentage of model sum of squares in the total sum of squares). A perfect fit would result in 100% rhythmicity. A *p*-value of below 0.05 was considered statistically significant and rhythmicity was considered robust above 30%.

## 3. Results

### 3.1. Clinical Parameters

Throughout the study, no deviations of the basal levels of the measured clinical parameters (rumen contractions, heart rate, rectal temperature and chewing activity) were observed.

### 3.2. Non-Esterified Fatty Acids

**Figure 1:**
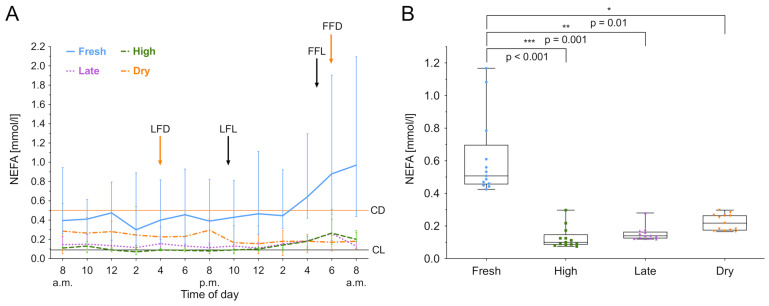
Non-esterified fatty acids (NEFA) concentration in serum over 24 h. (**A**) Data expressed as group median with the interquartile range as error bars. Last and first feeding of the day and cut off levels for group investigations [9] are shown. Black color indicates lactating cows, orange represents dry cows. LFL: last feeding of lactating cows; FFL: first feeding of lactating cows; LFD: last feeding of dry cows; FFD: first feeding of dry cows; CL: Cut off level of lactating cows; CD: Cut off level of dry cows. (**B**) Within-group distribution of NEFA concentration in serum over 24 h. The box depicts the mean and the lower and upper quartile. The whiskers show minimum and maximum values. Brackets represent Tukey adjusted pairwise comparisons between lactation group means.

**Figure 2:**
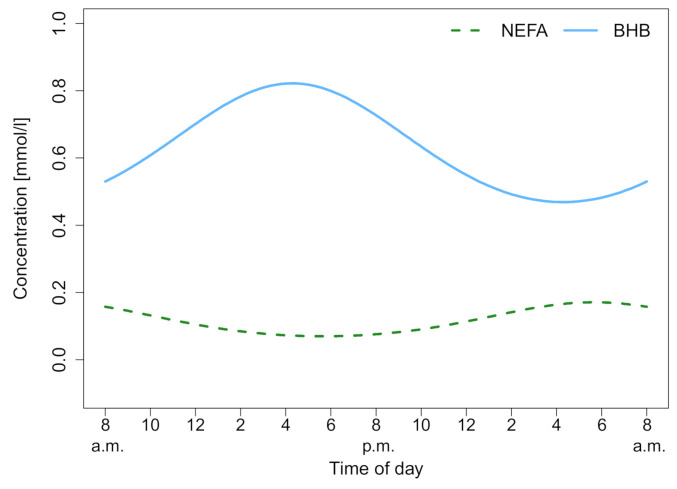
Cosine fits of non-esterified fatty acids (NEFA) and beta-hydroxybutyrate (BHB) for high lactation. Both show a significant circadian rhythm which can be described as a cosine function, (NEFA: *p* < 0.001, R^2^ = 45.96%; BHB: *p* < 0.001, R^2^ = 52.08%).

NEFA was measured over 24 h and the median biomarker level with the interquartile range (IQR) of the different lactation groups are shown in Figure 1A. The lactation phase had a significant effect on NEFA concentration ($\chi^{2}$(3) = 14.24, *p* = 0.003), as did the time ($\chi^{2}$(12) = 64.65, *p* < 0.001). Fresh lactation had an effect on the time course of NEFA (b = 1.41, *t*(12) = 4.31; *p* = 0.001). In dry cows, a steady NEFA level throughout the study period was observed. Lactating cows showed a NEFA increase during the night. After the last feeding (9.30 pm), NEFA concentrations increased in all lactating groups. For high and late lactating cows, the NEFA value at 6.00 am was higher than the individuals daily mean, *t*(7) = 3.96; *p* = 0.005. Furthermore, post hoc comparisons using Tukey’s test showed that fresh lactating cows have higher levels than high lactating (*p* < 0.001), late lactating (*p* = 0.001) and dry cows (*p* = 0.01). Furthermore, an increased within-group variation of NEFA in fresh lactating cows was observed (Figure 1B). High lactating cows exhibit circadian rhythmicity (*F*_8,43_ = 4.5711, *p* < 0.001), as shown in Figure 2, whereas the rhythm peaked at 5.47 am. Only high lactating cows showed a significant rhythmicity and the highest absolute values. The second largest rhythmicity was observed during late lactation, reaching a minimum in dry cows, and finally rising again in fresh lactating cows. A summary of the computed rhythmicity parameters is presented in Table 1.

### 3.3. Beta-Hydroxybutyrate

**Figure 3:**
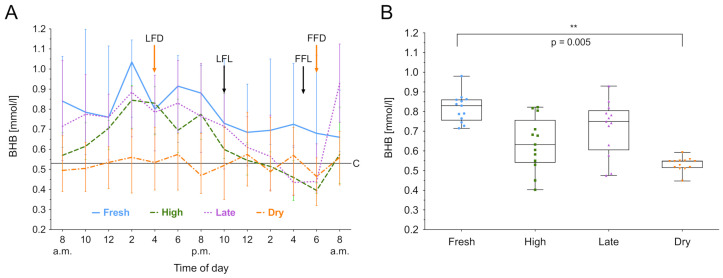
Beta-hydroxybutyrate (BHB) concentration in serum over 24 h. (**A**) Data expressed as group median with the interquartile range as error bars. Last and first feeding of the day and cut off level for group investigations [9] are shown. Black color indicates lactating cows, orange represents dry cows. LFL: last feeding of lactating cows; FFL: first feeding of lactating cows; LFD: last feeding of dry cows; FFD: first feeding of dry cows; C: Cut off level for all cows. (**B**) Within-group distribution of BHB concentration in serum over 24 h. The box depicts the median and the lower and upper quartile. The whiskers show minimum and maximum values. Bracket represent Tukey adjusted pairwise comparisons between lactation group means.

BHB was measured over 24 h, and the median biomarker level with the IQR of the different lactation groups are shown in Figure 1A. The lactation phase had a significant effect on BHB concentration ($\chi^{2}$(3) = 8.81, *p* = 0.03), as did the time ($\chi^{2}$(12) = 73.50, *p* < 0.001). For high and late lactating and dry cows, the BHB value at 6.00 am was lower than the individuals daily mean, *t*(11) = −4.60; *p* < 0.001. In fresh, high and late lactating cows the BHB value at 2.00 pm was higher than the individuals mean, *t*(11) = 5.17; *p* = 0.003. Post hoc comparisons using Tukey’s test showed that fresh lactating cows have higher levels than dry cows (*p* = 0.005). Furthermore, a decreased within-group variation of BHB in dry cows was noticed (Figure 1B). High lactating cows exhibit circadian rhythmicity (*F*_8,43_ = 5.8418, *p* < 0.001), as shown in Figure 3. Whereas the rhythm peaked at 4.20 pm, no significant effects in fresh, late lactating or dry cows were found. A summary of the computed rhythmicity parameters is presented in Table 1.

### 3.4. Total Bilirubin

**Figure 4:**
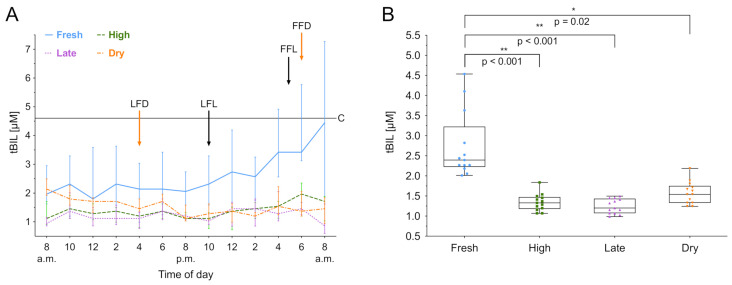
Total bilirubin (tBIL) concentration in serum over 24 h. (**A**) Data expressed as group median with the interquartile range as error bars. Last and first feeding of the day and cut off level for group investigations [9] are shown. Black color indicates lactating cows, orange represents dry cows. LFL: last feeding of lactating cows; FFL: first feeding of lactating cows; LFD: last feeding of dry cows; FFD: first feeding of dry cows; C: Cut off level for all cows. (**B**) Within-group distribution of tBIL concentration in serum over 24 h. The box depicts the median and the lower and upper quartile. The whiskers show minimum and maximum values. Brackets represent Tukey adjusted pairwise comparisons between lactation group means.

tBIL was measured over 24 h, and the median biomarker level with the IQR of the different lactation groups is shown in Figure 4A. The lactation phase had a significant effect on tBIL concentration ($\chi^{2}$(3) = 14.66, *p* < 0.01), as did the time ($\chi^{2}$(12) = 38.05, *p* < 0.001). Fresh lactation has an effect on the time course of tBIL (b = 0.63, *t*(12) = 3.81; *p* < 0.01). Additionally, post hoc comparisons using Tukey’s test showed that fresh lactating cows have higher levels than high lactating (*p* < 0.001), late lactating (*p* < 0.001) and dry cows (*p* = 0.02). Furthermore, an increased within-group variation of tBIL in fresh lactating cows was observed (Figure 4B). No circadian rhythmicity was found for tBIL. A summary of the computed rhythmicity parameters is presented in Table 1.

### 3.5. Aspartate Aminotransferase

**Figure 5:**
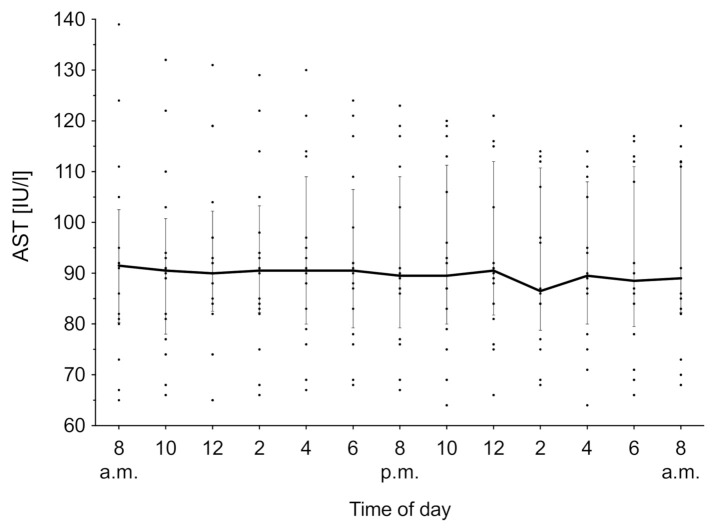
Aspartate aminotransferase (AST) concentration in serum over 24 h. Data expressed as median over all groups (*n* = 16) with the interquartile rage as error bars. Individual measurements are depicted as aligned dots.

AST was measured for 24 h, and the time course of the overall median biomarker level with IQR is shown in Figure 5. Both the lactation phase ($\chi^{2}$(3) = 4.70, *p* = 0.19) and time ($\chi^{2}$(12) = 6.79, *p* = 0.87) have no effect on the AST activity. The overall median was 92.38 IU/mL (IQR 91.72–92.84 IU/mL).

## 4. Discussion

This study investigated the effects of lactation phases and time on serum biomarker concentrations in 16 Simmental dairy cows by observational short-term monitoring (24 h). Both tested parameters influenced the concentration of the biomarkers NEFA, BHB and tBIL in serum. Moreover, the lactation phase effects the degree of circadian rhythmicity of biomarkers, wherein robust circadian rhythms were found in high lactating cows for NEFA and BHB.

Cows were only exposed to a minimum of external manipulations to avoid stress induced by the study protocol. Hence, it was possible to collect several samples on ruminating lying cows, enabling as few interferences as possible, since only 10% of samples needed more rigorous manipulations. To meet all sampling requirements for an optimal chronobiological study in a pilot study is hardly feasible. Cornelissen [25] describes the analysis of a hybrid (linked cross sectional) 24 h study design. Here serial measurements were collected from several individuals. The circadian rhythm of each individual was assessed by a single cosinor analysis and summarized across the group. Choosing a 4x4 study design allowed us a first insight in the variation of circadian rhythms across the four lactation phases under similar conditions. Indisputably, the larger the number of individuals per group, the better the representation the population. However, using four individuals per group allowed sample withdrawal of all cows in a very short time frame so that one group could be tested on the same day and time points and handled by the same team. Altogether, this ensured consistent conditions for individuals, limited manipulations like tethering the individuals and other variables to an absolute minimum. The reported range of cows age is quite wide (1st to 9th lactation). However, due to the maximum expected weight gain of 10% occurring from the 1st to the 2nd lactation, its effect on the investigated metabolic biomarkers is considerably low. Thus, our small data set solely reflect the influence of individual behavioral patterns, lactation phase and day time on biomarkers.

As anticipated, NEFA and BHB undergo postprandial changes in high and late lactating cows. NEFA exhibits a sharp increase after the last feed intake with a peak at 6:00 am followed by a decrease after the first feed intake. Rottman et al. [34] found in his work, on 17 high lactating cows investigating daily rhythm of milk synthesis in relation to feed supply, as well a peak of NEFA at 6:00 am directly followed by independent to the applied feeding treatment (1 time fed versus 4 times fed). He claims that NEFA are regulated not only by fed supply and that the observed decreases after the last feeding of the day can be explained by the constant energy demand for lactation, and thereby caused negative energy balance. Postprandial decreases are the result of insulin expression causing a down regulation of lipolysis [27]. A basal level, in terms of a physiological baseline, of NEFA was observed about two to three hours after the first feeding until shortly after the last feeding. For BHB a continuous decrease after the last feed intake and followed by a sharp increase directly after the first feeding was noticed. Here the observed postprandial increase is most likely accounted for by feedstuff induced ruminal ketogenesis and not by hepatic ketogenesis [26]. Microbial fermentation in the rumen leads to the production of short chain fatty acids (acetate, butyrate and propionate). Propionate triggers insulin expression [35] and butyrate is then further metabolized to BHB [26]. Furthermore, butyric acid is also a natural component of the feedstuff and partly metabolized to BHB. The exact amount of butyric acid in the feedstuff is unknown. However, only high-quality silages with low butyrate content were used in this study. A basal level of BHB was observed shortly before and after the first feeding. Those findings implicate that feed intakes and activity levels are most likely the determining factors for diurnal variations in NEFA and BHB in high and late lactating cows. Thus, elevated concentrations of the basal level could reveal pathological elevated body fat mobilization early on. It can be concluded that NEFA concentration in healthy cows is directly linked to the energy metabolism and it is elevated during anabolic phases [36]. In high and late lactating cows our data on diurnal variations are consistent with previous studies investigating 24 or 48 h patterns (numbers of individuals ranging from 6 to 16) on NEFA [27,37,38,39,40] and BHB [26,27,38,41].

On the contrary, fresh lactating cows exhibit a significantly different biomarker daily pattern. For NEFA and tBIL a rising trend was observed. No clear trend or circadian rhythmicity was found for BHB. These results are in agreement with Mahrt et al. [42]. The authors also didn’t find a time dependence for BHB in continuously fed fresh lactating cows (8–28 days in milk (DIM)). Especially cows in their transition period are known to be a high risk group for metabolic diseases [43]. Results of our study point in the same direction. Fresh lactating cows exhibit significantly higher levels of NEFA, BHB and tBIL compared to the other groups. Further, the group median NEFA concentration of fresh lactating cows (0.51 mmol/L) also drastically exceeds the control upper level for herd diagnostic of 0.09 mmol/L proposed by Fürll [9]. Additionally, the steadily increasing NEFA concentration and lack of the expected postprandial changes in NEFA and BHB demonstrate the metabolic challenges and instability of fresh lactating cows. A follow up study over an extended time period (e.g., 72 h) could reveal the interaction between external factors and metabolism in fresh cows.

In dry cows the NEFA concentration stayed at their basal level throughout the study period. The relative insulin resistance occurring in adipose tissue and skeletal muscle occurring during late pregnancy [12] and thereby reduced responsiveness of hepatic gluconeogenesis to available feed could explain the lack significant diurnal changes. Further, only minor diurnal variations were observed for BHB and tBIL. Similar results were reported previously by Wiedemann et al. [44].

Moreover, our results indicate that not only diurnal variations of biomarkers change throughout the lactation stages, but also their degree of circadian rhythmicity does. NEFA and BHB show significant and robust circadian rhythms in high lactating cows. Both variables reach their acrophase at a different time and vary in the height of the amplitude. In NEFA the peak level was about 50% higher than the basal level. Maximum BHB concentration was increased by a third. No robust circadian rhythmicity was found for all other lactation stages. The physiology suggests that circadian rhythmicity might be masked by metabolic challenges such as late term pregnancy and early lactation. This is also supported by Giannetto and Piccione [29], who found a robust (86.80%) circadian rhythm for NEFA in non-pregnant and non-lactating cows. Further, metabolic diseases such as fat cow syndrome and subclinical ketosis could influence circadian rhythms, since the liver plays a crucial role in the energy metabolism and is mainly responsible for an adequate feed response. Also, a mechanistic link between metabolism and circadian rhythms in the liver has been already established by Rutter et al. [23].

As noted before, no effect of time and lactation phase was observed in AST. Diagnostically, AST typically serves as marker for liver cell integrity, and an increased serum activity is indicative of tissue damage [9]. Similar results were found by Wiedemann et al. [44]. The authors suggest that the lack of diurnal variations might also be due to its long enzyme half-life. No connection to metabolic pathways or other external zeitgebers could be identified.

As mentioned earlier previous studies already suggest that biomarkers such as NEFA, BHB are strongly influenced by the feeding time and frequency [28,45], and show within day variations [41,44]. Mahrt et al. [42] found no time dependency for BHB. The authors further proposed that the observed time effects are mainly provoked by the applied feeding regimes. However, our results indicate that the NEFA, BHB and tBIL concentrations are not only affected by time but are also strongly influenced by the lactation phase. Thus, contradicting findings of previous studies could be a result of different enrollment criteria and study protocols, e.g., lactation phases, feeding strategies, changes in housing and activity levels.

Furthermore, we propose that it is possible to determine if the individual’s energy demand is met by intra-day monitoring of NEFA. The high diurnal variation of biomarkers is a main problem for single-point measurements. As mentioned earlier, the biomarker peak concentration can reach up to 50% of the basal level. Additionally, in practice exact monitoring of individuals behavior and calculating their energy balance according to the guidelines of the Society of Nutrition Physiology (GfE) [46] is not feasible. Our data suggest a strong link between feeding and day time and intraday NEFA variation. Introducing an intra-day monitoring of NEFA could thereby provide a deeper insight about the individual’s energy metabolism. We suggest measuring the feedback of NEFA to last and first feedings. The first measurement should be scheduled shortly after the last feeding of the day (I), followed by the second shortly after the first feeding of the following day (II), and the third two hours after the second measurement (III). An evaluation of the leaps after the last feeding (II-I) and after the third measurement (III-II) could reveal information about individual’s energy coverage. NEFA and BHB are already commonly used to determine a low energy status and negative energy balance [28,45]. Our proposed three-point measurement regime of NEFA could serve as a more direct and reliable source of information.

Although this pilot study was conducted only on a limited number of cows of a single breed with a moderate milk yield for a time frame of 24 h, we strongly emphasize that the interaction of lactation phase and sampling time should be considered for metabolic biomarker guidelines and health monitoring. Furthermore, we want to highlight the variance and resulting uncertainty of a single time point measurement in diagnostics. Especially in threshold diseases, diurnal variations and circadian rhythms have a profound impact on accurate diagnosis and help avoiding false positive and false negative test results. We recommend follow up studies in a larger scale, a more extended time period of 48 h and preferably for the studies to be repeated over several days to build a stronger representation of the population and investigate day to day variability.

## 5. Conclusions

This study investigated the effect of lactation phases on diurnal variations. We found that the diurnal variation and circadian rhythmicity is highly influenced by the lactation phase. Therefore, within veterinary practice, we suggest blood sampling within three hours after the first feeding until two hours after the last feeding of the day in order to prevent possible variations due to circadian rhythms.

## Figures and Tables

**Table 1:** Parameters of circadian rhythmicity for non-esterified fatty acids (NEFA), beta-hydroxybutyrate (BHB), and total bilirubin (tBIL). MESOR: Midline Estimating Statistic Of Rhythm or rhythm-adjusted mean. Significant cosine fits are highlighted in bold.

Biomarker	Lactation-phase	MESOR ± SE	Amplitude ± SE	Acrophase	*p*-Value	Rhythmicity
NEFA [mmol/L]	Dry	0.18 ± 0.02	0.06 ± 0.01	01:40 pm	0.89	7.57%
	Fresh	0.45 ± 0.05	0.22 ± 0.03	05:08 am	0.56	13.66%
	**High**	**0.11 ± 0.01**	**0.06 ± 0.00**	**05:39 A.M.**	**<0.001**	**45.96%**
	Late	0.14 ± 0.01	0.03 ± 0.00	06:32 am	0.29	20.60%
BHB [mmol/L]	Dry	0.51 ± 0.02	0.01 ± 0.00	07:23 pm	1.00	0.40%
	Fresh	0.79 ± 0.03	0.07 ± 0.00	03:57 pm	0.96	5.53%
	**High**	**0.62 ± 0.02**	**0.20 ± 0.01**	**04:20 pm**	**<0.001**	**52.08%**
	Late	0.69 ± 0.03	0.16 ± 0.01	03:07 pm	0.22	20.73%
tBIL [µmol/L]	Dry	1.51 ± 0.05	0.27 ± 0.01	11:16 am	0.23	20.23%
	Fresh	2.38 ± 0.16	0.76 ± 0.07	04:45 am	0.47	15.39%
	High	1.27 ± 0.05	0.19 ± 0.01	07:07 am	0.71	11.11%
	Late	1.19 ± 0.05	0.06 ± 0.00	01:41 am	1.00	1.36%

## Data Availability

The original contributions generated for the study are included in the article; further inquiries can be directed to the corresponding author.
